# 2,2′-{(1*E*,1′*E*)-[Ethane-1,2-diylbis(aza­nylyl­idene)]bis­(methanylyl­idene)}bis­(4-iodo­phenol)

**DOI:** 10.1107/S2414314622008951

**Published:** 2022-09-13

**Authors:** Lauren A. Blackwelder, Andrea R. Kelley, Gary J. Balaich, Latisha R. Jefferies

**Affiliations:** a Department of Chemistry, US Air Force Academy, CO, 80840, USA; Dublin City University, Ireland

**Keywords:** crystal structure, Schiff base, hydrogen bonding, π–π stacking

## Abstract

The title compound, C_20_H_14_I_2_N_2_O_2_, a di­iodo-Schiff base, crystallizes in space group *Pbca* with one mol­ecule per asymmetric unit. The mol­ecular structure reveals two intra­molecular O—H⋯N hydrogen bonds that give the mol­ecule a twisted structure with non-coplanar rings. In the crystal structure, the mol­ecular packing is stabilized by π–π stacking, hydrogen- and halogen-bonding (C—H⋯I; O⋯I) inter­actions.

## Abstract 2

The title compound is an di­iodo Schiff base mol­ecule under investigation for possible anti­microbial activity as well as for a ligand for medicinal activity.

## Graphical abstract

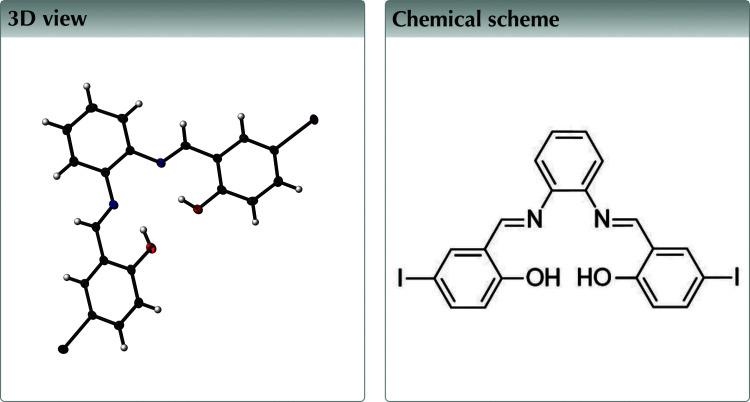

## Structure description

**Figure 1:**
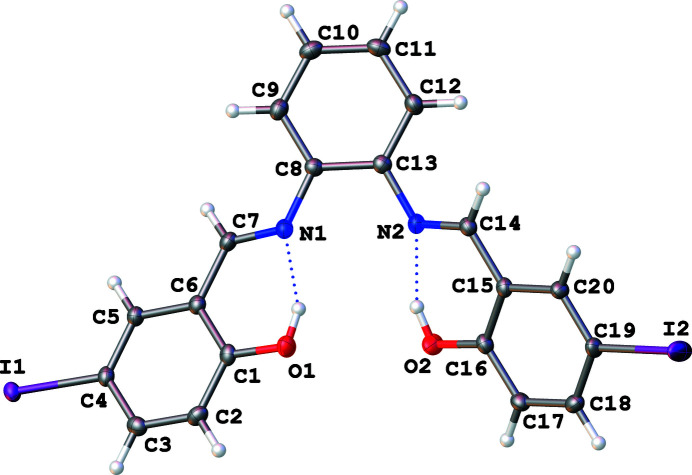
Molecular structure of (I) depicting the two intramolecular hydrogen bonds (O1—H1⋯N1 and O2—H2⋯N2). Displacement ellipsoids are shown at the 50% probability level

2,2′-{(1*E*,1′*E*)-[Ethane-1,2-diylbis(aza­nylyl­idene)]bis­(methanylyl­idene)}bis­(4-iodo­phen­ol) (I) (C_20_H_14_I_2_N_2_O_2_, Fig. 1 ▸) is a salen-type ligand under investigation in our laboratory for possible anti­microbial effects (Ceramella *et al.*, 2022 ▸) and the ability to bind to lanthanide metals, which could have other important medicinal properties (Kaczmarek *et al.*, 2018 ▸). As a result of the wide range of medical applications for such compounds, and in a continuation of our work in this area (Reimann *et al.*, 2019 ▸), the title compound (I) was prepared and its crystal structure is reported here.

**Figure 2:**
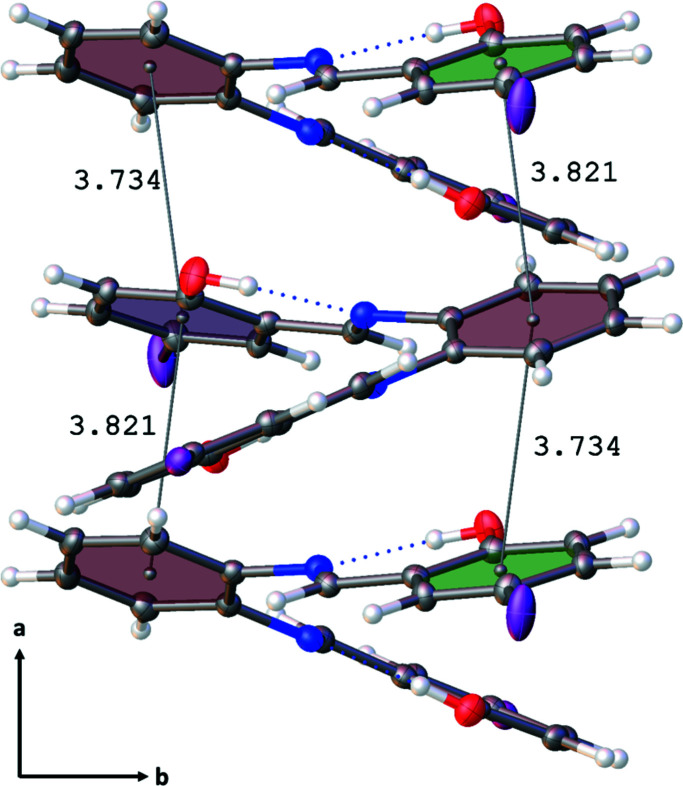
Diagram of the basic packing motif along the *a*-axis direction depicting the slipped ring π–π inter­actions.

**Figure 3:**
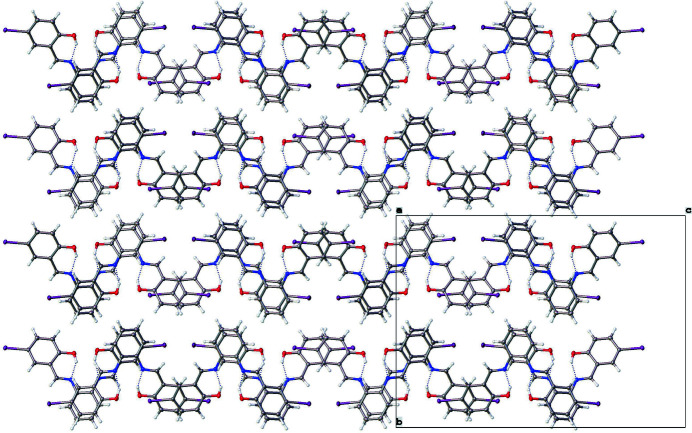
Mol­ecular packing view of (I) along the *a*-axis direction. Displacement ellipsoids are shown at the 50% probability level.

Each mol­ecule of (I) consists of a central ring (C8–C13) with *ortho* imine nitro­gen atoms (N1 and N2) covalently bound through the imine C atoms, C7 and C14 respectively, to I1—Ar(O1—H1) and I2—Ar(O2—H2) rings (Fig. 1 ▸). Two intra­molecular hydrogen bonds (Table 1 ▸), O1—H1⋯N1 [1.842 (15) Å, 152 (3)°] and O2—H2⋯N2 [1.806 (15) Å, 153 (3)°], result in an overall non-planar mol­ecule with the I—Ar(OH) ring planes twisted with respect to the central ring (C8–13) plane [24.97 (7)° *versus* I1—Ar(O1—H1), and 39.37 (5)° *versus* I2—Ar(O2—H2)] (Fig. 1 ▸). The intra­molecular hydrogen bonds of (I) show similarity to those of the dichlorinated Schiff base 3,5-di­chloro-*N*-[2-(methyl­thio)­phen­yl]salicylaldimine (Hamaker *et al.*, 2010 ▸). Halogen bonding (I1⋯O2) and slipped π–π stacking inter­actions stabilize the packing pattern. Along the *a*-axis direction, adjacent mol­ecules related by 2_1_ screw-axis symmetry form 2-stacks/mol­ecule of non-coplanar and alternating central ring (C8–C13) to I2—Ar(O2—H2) ring inter­actions (Fig. 2 ▸). This results in alternating longer [3.821 (13) Å] and shorter [3.734 (13) Å] central ring (C8–C13) centroid to I2—Ar(O2—H2) ring centroid distances with the corresponding alternating centroids slipped by 1.583 (4) and 1.548 (3) Å with respect to each other (Fig. 2 ▸). The additional hydrogen bonding provides three shorter and one longer C—H⋯I type inter­actions in which I2 has a larger displacement ellipsoid than I1 [C18—H18⋯I1 = 3.16 Å (159°), C5—H5⋯I2 = 3.17 Å (156°), C7—H7⋯I2 = 3.23 Å (152°), and C2—H2⋯I2 3.32 Å (131°)]. The two C—I bond lengths of (I) are similar to the C—I bond of 2,3,5,6-tetra­fluoro-1,4-di­iodo­benzene (Tan & Tiekink, 2019 ▸). Along the *b*-axis direction, adjacent mol­ecules appear to be arranged in a H (head, central C8–C13 ring) to T [tail, I1—Ar(O1—H1) and I2—Ar(O2—H2) rings] repeating pattern with the I1 and I2 atoms of the I1—Ar(O1—H1) and I2—Ar(O2—H2) rings inter­digitated along the *c*-axis direction (Fig. 3 ▸).

## Synthesis and crystallization

To a solution of 2-hy­droxy-5-iodo­benzaldehyde (4.60 g, 18.5 mmol) in ethanol (200 ml) was added an ethanol (10 ml) solution of *o*-phenyl­enedi­amine (1.00 g, 9.20 mmol), and the reaction mixture brought to reflux with vigorous stirring for 2 h. Upon cooling, the title compound (I) precipitated as an orange solid, and was filtered, washed with ethanol and dried under vacuum. Crystals of (I) suitable for single-crystal X-ray diffraction were grown from acetone layered with hexane. Yield: 4.37 g (83%), m.p. 212–214°C. ^1^H NMR (500 MHz, CDCl_3_) δ 8.54 (*s*, 2H), 7.73–7.54 (*m*, 4H), 7.37 (*q*, *J* = 3.2 Hz, 2H), 7.22 (*q*, *J* = 3.2 Hz, 2H), 6.84 (*d*, *J* = 9.0 Hz, 2H). [Note: phenolic H atoms (2Hs) undergo rapid exchange thus their NMR signals are broadened into the baseline beyond recognition as reported (Charisiadis *et al*., 2014 ▸).] ^13^C NMR (500 MHz, CDCl_3_) δ 162.30, 161.11, 142.25, 141.81, 140.46, 128.32, 121.46, 120.15, 119.63, 79.69. MALDI–TOF MS: monoisotopic *m*/*z* calculated for [*M* + H]^+^: 568.9; observed: 568.8.

## Refinement

Crystal data, data collection and structure refinement details of (I) are summarized in Table 2 ▸.

## Supplementary Material

Crystal structure: contains datablock(s) I. DOI: 10.1107/S2414314622008951/gg4010sup1.cif

Structure factors: contains datablock(s) I. DOI: 10.1107/S2414314622008951/gg4010Isup2.hkl

CCDC reference: 2205660

Additional supporting information:  crystallographic information; 3D view; checkCIF report

## Figures and Tables

**Table 1:** Hydrogen-bond geometry (Å, °)

*D*—H⋯*A*	*D*—H	H⋯*A*	*D*⋯*A*	*D*—H⋯*A*
C2—H2*A*⋯I2^i^	0.95	3.32	4.009 (2)	131
C5—H5⋯I2^ii^	0.95	3.17	4.061 (2)	156
C7—H7⋯I2^ii^	0.95	3.23	4.094 (2)	152
C18—H18⋯I1^iii^	0.95	3.16	4.066 (2)	159
O1—H1⋯N1	0.84 (1)	1.84 (2)	2.609 (3)	152 (3)
O2—H2⋯N2	0.84 (1)	1.81 (2)	2.580 (3)	153 (3)

Symmetry codes: (i) 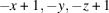 ; (ii) 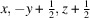 ; (iii) 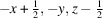 .

**Table 2:** Experimental details

Crystal data	
Chemical formula	C_20_H_14_I_2_N_2_O_2_
*M*_r_	568.13
Crystal system, space group	Orthorhombic, *P* *b* *c* *a*
Temperature (K)	112
*a*, *b*, *c* (Å)	7.4776 (1), 19.1040 (2), 26.0919 (3)
*V* (Å^3^)	3727.28 (8)
*Z*	8
Radiation type	Mo *K*α
μ (mm^−1^)	3.39
Crystal size (mm)	0.30 × 0.26 × 0.12

Data collection	
Diffractometer	XtaLAB Synergy, Single source at offset/far, HyPix3000
Absorption correction	Gaussian (*CrysAlis PRO*; Rigaku OD, 2019 ▸)
*T*_min_, *T*_max_	0.300, 1.000
No. of measured, independent and observed [*I* > 2σ(*I*)] reflections	34066, 4062, 3774
*R*_int_	0.033
(sin θ/λ)_max_ (Å^−1^)	0.648

Refinement	
*R*[*F* ^2^ > 2σ(*F* ^2^)], *wR*(*F* ^2^), *S*	0.020, 0.044, 1.09
No. of reflections	4062
No. of parameters	242
No. of restraints	2
H-atom treatment	H atoms treated by a mixture of independent and constrained refinement
Δρ_max_, Δρ_min_ (e Å^−3^)	0.66, −0.92

Computer programs: *CrysAlis PRO* (Rigaku OD, 2019 ▸), *SHELXT2014/5* (Sheldrick, 2015*a* ▸), *SHELXL* (Sheldrick, 2015*b* ▸), and *OLEX2* (Dolomanov *et al.*, 2009 ▸).

## full crystallographic data

### Crystal data

**Table:**

C_20_H_14_I_2_N_2_O_2_	*D*_x_ = 2.025 Mg m^−^^3^
*M**_r_* = 568.13	Mo *K*α radiation, λ = 0.71073 Å
Orthorhombic, *P**b**c**a*	Cell parameters from 25879 reflections
*a* = 7.4776 (1) Å	θ = 1.5–27.4°
*b* = 19.1040 (2) Å	µ = 3.39 mm^−^^1^
*c* = 26.0919 (3) Å	*T* = 112 K
*V* = 3727.28 (8) Å^3^	Rect. Prism, orange
*Z* = 8	0.30 × 0.26 × 0.12 mm
*F*(000) = 2160	

### Data collection

**Table:**

XtaLAB Synergy, Single source at offset/far, HyPix3000 diffractometer	4062 independent reflections
Radiation source: micro-focus sealed X-ray tube, PhotonJet (Mo) X-ray Source	3774 reflections with *I* > 2σ(*I*)
Mirror monochromator	*R*_int_ = 0.033
Detector resolution: 10.0000 pixels mm^-1^	θ_max_ = 27.4°, θ_min_ = 1.6°
ω scans	*h* = −9→9
Absorption correction: gaussian (CrysAlisPro; Rigaku OD, 2019)	*k* = −23→24
*T*_min_ = 0.300, *T*_max_ = 1.000	*l* = −32→32
34066 measured reflections	

### Refinement

**Table:**

Refinement on *F*^2^	Hydrogen site location: mixed
Least-squares matrix: full	H atoms treated by a mixture of independent and constrained refinement
*R*[*F*^2^ > 2σ(*F*^2^)] = 0.020	*w* = 1/[σ^2^(*F*_o_^2^) + (0.0133*P*)^2^ + 5.3948*P*] where *P* = (*F*_o_^2^ + 2*F*_c_^2^)/3
*wR*(*F*^2^) = 0.044	(Δ/σ)_max_ = 0.002
*S* = 1.09	Δρ_max_ = 0.66 e Å^−^^3^
4062 reflections	Δρ_min_ = −0.92 e Å^−^^3^
242 parameters	Extinction correction: SHELXL-2016/6 (Sheldrick 2015b, Fc^*^=kFc[1+0.001xFc^2^λ^3^/sin(2θ)]^-1/4^
2 restraints	Extinction coefficient: 0.000120 (17)
Primary atom site location: dual	

### Special details

**Table:**

Geometry. All esds (except the esd in the dihedral angle between two l.s. planes) are estimated using the full covariance matrix. The cell esds are taken into account individually in the estimation of esds in distances, angles and torsion angles; correlations between esds in cell parameters are only used when they are defined by crystal symmetry. An approximate (isotropic) treatment of cell esds is used for estimating esds involving l.s. planes.

### Fractional atomic coordinates and isotropic or equivalent isotropic displacement parameters (Å^2^)

**Table:**

	*x*	*y*	*z*	*U*_iso_*/*U*_eq_	
I1	0.48524 (2)	0.12444 (2)	0.85138 (2)	0.01709 (5)	
I2	0.28304 (3)	0.11138 (2)	0.30070 (2)	0.03584 (7)	
O1	0.4758 (2)	0.15083 (9)	0.61326 (6)	0.0205 (4)	
O2	0.1200 (2)	0.13607 (9)	0.53361 (6)	0.0233 (4)	
N1	0.3343 (3)	0.27475 (10)	0.62428 (7)	0.0162 (4)	
N2	0.1963 (3)	0.26760 (10)	0.52825 (7)	0.0163 (4)	
C1	0.4718 (3)	0.14643 (12)	0.66467 (9)	0.0159 (5)	
C2	0.5299 (3)	0.08468 (12)	0.68798 (9)	0.0183 (5)	
H2A	0.569184	0.046666	0.667387	0.022*	
C3	0.5307 (3)	0.07839 (12)	0.74077 (9)	0.0181 (5)	
H3	0.571539	0.036324	0.756275	0.022*	
C4	0.4721 (3)	0.13336 (12)	0.77109 (9)	0.0158 (5)	
C5	0.4097 (3)	0.19424 (12)	0.74922 (9)	0.0162 (5)	
H5	0.367238	0.231184	0.770347	0.019*	
C6	0.4087 (3)	0.20174 (12)	0.69545 (8)	0.0154 (5)	
C7	0.3358 (3)	0.26551 (12)	0.67317 (8)	0.0167 (5)	
H7	0.288625	0.300786	0.694995	0.020*	
C8	0.2667 (3)	0.33732 (12)	0.60266 (8)	0.0152 (5)	
C9	0.2710 (3)	0.40210 (13)	0.62775 (9)	0.0190 (5)	
H9	0.316488	0.405051	0.661683	0.023*	
C10	0.2096 (3)	0.46185 (12)	0.60350 (9)	0.0201 (5)	
H10	0.212521	0.505533	0.620872	0.024*	
C11	0.1437 (3)	0.45812 (12)	0.55393 (9)	0.0199 (5)	
H11	0.100415	0.499165	0.537540	0.024*	
C12	0.1408 (3)	0.39464 (12)	0.52821 (9)	0.0192 (5)	
H12	0.095597	0.392368	0.494231	0.023*	
C13	0.2040 (3)	0.33413 (12)	0.55195 (8)	0.0154 (5)	
C14	0.2275 (3)	0.26073 (12)	0.47991 (8)	0.0161 (5)	
H14	0.260311	0.300477	0.460144	0.019*	
C15	0.2129 (3)	0.19260 (12)	0.45542 (8)	0.0156 (5)	
C16	0.1605 (3)	0.13285 (12)	0.48342 (9)	0.0165 (5)	
C17	0.1499 (3)	0.06804 (12)	0.45880 (9)	0.0180 (5)	
H17	0.115967	0.027658	0.477692	0.022*	
C18	0.1884 (3)	0.06224 (12)	0.40725 (9)	0.0186 (5)	
H18	0.181371	0.018057	0.390663	0.022*	
C19	0.2375 (3)	0.12147 (12)	0.37979 (9)	0.0183 (5)	
C20	0.2510 (3)	0.18604 (12)	0.40303 (8)	0.0168 (5)	
H20	0.285889	0.225866	0.383654	0.020*	
H1	0.428 (3)	0.1892 (8)	0.6061 (10)	0.020*	
H2	0.139 (3)	0.1776 (7)	0.5419 (9)	0.020*	

### Atomic displacement parameters (Å^2^)

**Table:**

	*U*^11^	*U*^22^	*U*^33^	*U*^12^	*U*^13^	*U*^23^
I1	0.01938 (8)	0.01833 (8)	0.01356 (8)	−0.00151 (6)	−0.00173 (6)	0.00283 (5)
I2	0.07494 (16)	0.01979 (9)	0.01279 (9)	0.00557 (9)	0.00604 (8)	−0.00114 (6)
O1	0.0249 (9)	0.0219 (9)	0.0145 (8)	0.0034 (7)	0.0003 (7)	−0.0015 (7)
O2	0.0358 (10)	0.0204 (9)	0.0136 (8)	−0.0054 (8)	0.0043 (7)	0.0029 (7)
N1	0.0167 (10)	0.0177 (10)	0.0142 (9)	−0.0021 (8)	−0.0008 (8)	0.0004 (8)
N2	0.0172 (10)	0.0182 (10)	0.0135 (9)	−0.0003 (8)	−0.0012 (8)	−0.0005 (8)
C1	0.0121 (11)	0.0218 (12)	0.0138 (11)	−0.0023 (9)	−0.0012 (9)	−0.0008 (9)
C2	0.0162 (11)	0.0177 (12)	0.0211 (12)	0.0006 (9)	0.0006 (9)	−0.0046 (9)
C3	0.0149 (11)	0.0180 (12)	0.0215 (12)	0.0000 (9)	−0.0022 (9)	0.0022 (10)
C4	0.0161 (11)	0.0187 (11)	0.0127 (11)	−0.0035 (9)	−0.0009 (9)	0.0001 (9)
C5	0.0167 (11)	0.0181 (11)	0.0138 (10)	−0.0004 (10)	−0.0006 (9)	−0.0010 (9)
C6	0.0145 (11)	0.0170 (11)	0.0148 (11)	−0.0020 (9)	−0.0013 (9)	−0.0004 (9)
C7	0.0180 (11)	0.0171 (11)	0.0150 (11)	−0.0018 (9)	−0.0005 (9)	−0.0033 (9)
C8	0.0152 (11)	0.0155 (11)	0.0149 (11)	0.0001 (9)	0.0018 (9)	0.0008 (9)
C9	0.0203 (12)	0.0209 (12)	0.0157 (11)	−0.0029 (10)	0.0003 (9)	−0.0025 (10)
C10	0.0192 (12)	0.0163 (11)	0.0248 (12)	−0.0009 (10)	0.0035 (10)	−0.0042 (10)
C11	0.0195 (12)	0.0161 (11)	0.0241 (12)	0.0030 (10)	0.0025 (10)	0.0041 (10)
C12	0.0205 (12)	0.0206 (12)	0.0165 (11)	0.0016 (10)	−0.0017 (10)	0.0020 (9)
C13	0.0148 (11)	0.0165 (11)	0.0149 (11)	−0.0004 (9)	0.0020 (9)	0.0010 (9)
C14	0.0163 (11)	0.0170 (11)	0.0150 (11)	−0.0010 (9)	−0.0016 (9)	0.0023 (9)
C15	0.0147 (11)	0.0186 (11)	0.0135 (11)	−0.0010 (9)	−0.0017 (9)	0.0006 (9)
C16	0.0165 (11)	0.0193 (12)	0.0136 (11)	−0.0004 (9)	−0.0006 (9)	0.0029 (9)
C17	0.0196 (12)	0.0165 (11)	0.0179 (11)	−0.0027 (10)	−0.0009 (10)	0.0036 (9)
C18	0.0202 (12)	0.0157 (11)	0.0199 (12)	−0.0005 (9)	−0.0027 (10)	−0.0017 (9)
C19	0.0244 (12)	0.0188 (12)	0.0117 (11)	0.0023 (10)	0.0005 (9)	−0.0001 (9)
C20	0.0215 (12)	0.0151 (11)	0.0138 (11)	−0.0014 (9)	0.0009 (9)	0.0015 (9)

### Geometric parameters (Å, º)

**Table:**

I1—C4	2.104 (2)	C8—C9	1.401 (3)
I2—C19	2.100 (2)	C8—C13	1.405 (3)
O1—C1	1.344 (3)	C9—H9	0.9500
O1—H1	0.835 (10)	C9—C10	1.384 (3)
O2—C16	1.345 (3)	C10—H10	0.9500
O2—H2	0.836 (10)	C10—C11	1.386 (3)
N1—C7	1.288 (3)	C11—H11	0.9500
N1—C8	1.415 (3)	C11—C12	1.386 (3)
N2—C13	1.415 (3)	C12—H12	0.9500
N2—C14	1.290 (3)	C12—C13	1.394 (3)
C1—C2	1.397 (3)	C14—H14	0.9500
C1—C6	1.409 (3)	C14—C15	1.454 (3)
C2—H2A	0.9500	C15—C16	1.411 (3)
C2—C3	1.383 (3)	C15—C20	1.402 (3)
C3—H3	0.9500	C16—C17	1.397 (3)
C3—C4	1.386 (3)	C17—H17	0.9500
C4—C5	1.377 (3)	C17—C18	1.380 (3)
C5—H5	0.9500	C18—H18	0.9500
C5—C6	1.410 (3)	C18—C19	1.389 (3)
C6—C7	1.456 (3)	C19—C20	1.378 (3)
C7—H7	0.9500	C20—H20	0.9500
			
C1—O1—H1	105.6 (18)	C9—C10—C11	120.2 (2)
C16—O2—H2	104.9 (18)	C11—C10—H10	119.9
C7—N1—C8	120.9 (2)	C10—C11—H11	119.9
C14—N2—C13	120.8 (2)	C10—C11—C12	120.1 (2)
O1—C1—C2	118.7 (2)	C12—C11—H11	119.9
O1—C1—C6	122.0 (2)	C11—C12—H12	119.8
C2—C1—C6	119.3 (2)	C11—C12—C13	120.3 (2)
C1—C2—H2A	119.7	C13—C12—H12	119.8
C3—C2—C1	120.6 (2)	C8—C13—N2	117.7 (2)
C3—C2—H2A	119.7	C12—C13—N2	122.5 (2)
C2—C3—H3	120.0	C12—C13—C8	119.7 (2)
C2—C3—C4	120.1 (2)	N2—C14—H14	119.8
C4—C3—H3	120.0	N2—C14—C15	120.5 (2)
C3—C4—I1	119.49 (17)	C15—C14—H14	119.8
C5—C4—I1	119.79 (17)	C16—C15—C14	121.2 (2)
C5—C4—C3	120.7 (2)	C20—C15—C14	119.6 (2)
C4—C5—H5	120.0	C20—C15—C16	119.3 (2)
C4—C5—C6	120.0 (2)	O2—C16—C15	122.0 (2)
C6—C5—H5	120.0	O2—C16—C17	118.4 (2)
C1—C6—C5	119.3 (2)	C17—C16—C15	119.6 (2)
C1—C6—C7	121.7 (2)	C16—C17—H17	119.8
C5—C6—C7	119.0 (2)	C18—C17—C16	120.5 (2)
N1—C7—C6	120.9 (2)	C18—C17—H17	119.8
N1—C7—H7	119.6	C17—C18—H18	120.2
C6—C7—H7	119.6	C17—C18—C19	119.5 (2)
C9—C8—N1	123.5 (2)	C19—C18—H18	120.2
C9—C8—C13	119.1 (2)	C18—C19—I2	118.35 (17)
C13—C8—N1	117.3 (2)	C20—C19—I2	120.16 (17)
C8—C9—H9	119.7	C20—C19—C18	121.5 (2)
C10—C9—C8	120.5 (2)	C15—C20—H20	120.2
C10—C9—H9	119.7	C19—C20—C15	119.6 (2)
C9—C10—H10	119.9	C19—C20—H20	120.2
			
I1—C4—C5—C6	176.96 (17)	C8—N1—C7—C6	−178.7 (2)
I2—C19—C20—C15	177.30 (17)	C8—C9—C10—C11	−0.3 (4)
O1—C1—C2—C3	178.9 (2)	C9—C8—C13—N2	−178.1 (2)
O1—C1—C6—C5	−179.3 (2)	C9—C8—C13—C12	−2.5 (3)
O1—C1—C6—C7	3.2 (3)	C9—C10—C11—C12	−0.7 (4)
O2—C16—C17—C18	179.4 (2)	C10—C11—C12—C13	0.0 (4)
N1—C8—C9—C10	177.2 (2)	C11—C12—C13—N2	177.0 (2)
N1—C8—C13—N2	6.2 (3)	C11—C12—C13—C8	1.6 (3)
N1—C8—C13—C12	−178.1 (2)	C13—N2—C14—C15	−177.7 (2)
N2—C14—C15—C16	1.5 (3)	C13—C8—C9—C10	1.8 (3)
N2—C14—C15—C20	−178.7 (2)	C14—N2—C13—C8	−145.5 (2)
C1—C2—C3—C4	0.6 (4)	C14—N2—C13—C12	38.9 (3)
C1—C6—C7—N1	−3.3 (3)	C14—C15—C16—O2	0.7 (4)
C2—C1—C6—C5	1.6 (3)	C14—C15—C16—C17	−179.2 (2)
C2—C1—C6—C7	−176.0 (2)	C14—C15—C20—C19	179.9 (2)
C2—C3—C4—I1	−177.29 (17)	C15—C16—C17—C18	−0.7 (4)
C2—C3—C4—C5	1.1 (3)	C16—C15—C20—C19	−0.3 (3)
C3—C4—C5—C6	−1.5 (3)	C16—C17—C18—C19	−0.2 (4)
C4—C5—C6—C1	0.1 (3)	C17—C18—C19—I2	−177.10 (17)
C4—C5—C6—C7	177.7 (2)	C17—C18—C19—C20	0.8 (4)
C5—C6—C7—N1	179.2 (2)	C18—C19—C20—C15	−0.6 (4)
C6—C1—C2—C3	−1.9 (3)	C20—C15—C16—O2	−179.1 (2)
C7—N1—C8—C9	29.0 (3)	C20—C15—C16—C17	1.0 (3)
C7—N1—C8—C13	−155.5 (2)		

### Hydrogen-bond geometry (Å, º)

**Table:**

*D*—H···*A*	*D*—H	H···*A*	*D*···*A*	*D*—H···*A*
C2—H2*A*···I2^i^	0.95	3.32	4.009 (2)	131
C5—H5···I2^ii^	0.95	3.17	4.061 (2)	156
C7—H7···I2^ii^	0.95	3.23	4.094 (2)	152
C18—H18···I1^iii^	0.95	3.16	4.066 (2)	159
O1—H1···N1	0.84 (1)	1.84 (2)	2.609 (3)	152 (3)
O2—H2···N2	0.84 (1)	1.81 (2)	2.580 (3)	153 (3)

Symmetry codes: (i) −*x*+1, −*y*, −*z*+1; (ii) *x*, −*y*+1/2, *z*+1/2; (iii) −*x*+1/2, −*y*, *z*−1/2.
